# High‐Performance Organic–Inorganic Hybrid Conductive Hydrogels for Stretchable Elastic All‐Hydrogel Supercapacitors and Flexible Self‐Powered Integrated Systems

**DOI:** 10.1002/advs.202403358

**Published:** 2024-07-08

**Authors:** Tao Cheng, Zhong‐Ting Liu, Jie Qu, Chao‐Fu Meng, Ling‐Jun He, Lang Li, Xuan‐Li Yang, Yu‐Jie Cao, Kai Han, Yi‐Zhou Zhang, Wen‐Yong Lai

**Affiliations:** ^1^ State Key Laboratory of Organic Electronics and Information Displays (SKLOEID) Institute of Advanced Materials (IAM) School of Chemistry and Life Sciences Nanjing University of Posts & Telecommunications 9 Wenyuan Road Nanjing 210023 China; ^2^ Institute of Advanced Materials and Flexible Electronics (IAMFE) School of Chemistry and Materials Science Nanjing University of Information Science & Technology Nanjing 210044 China

**Keywords:** confinement self‐assembly, flexible energy storage devices, flexible self‐powered integrated systems, flexible/wearable electronics, organic–inorganic hybrid conductive hydrogels

## Abstract

Conductive polymer hydrogels exhibit unique electrical, electrochemical, and mechanical properties, making them highly competitive electrode materials for stretchable high‐capacity energy storage devices for cutting‐edge wearable electronics. However, it remains extremely challenging to simultaneously achieve large mechanical stretchability, high electrical conductivity, and excellent electrochemical properties in conductive polymer hydrogels because introducing soft insulating networks for improving stretchability inevitably deteriorates the connectivity of rigid conductive domain and decreases the conductivity and electrochemical activity. This work proposes a distinct confinement self‐assembly and multiple crosslinking strategy to develop a new type of organic–inorganic hybrid conductive hydrogels with biphase interpenetrating cross‐linked networks. The hydrogels simultaneously exhibit high conductivity (2000 S m^−1^), large stretchability (200%), and high electrochemical activity, outperforming existing conductive hydrogels. The inherent mechanisms for the unparalleled comprehensive performances are thoroughly investigated. Elastic all‐hydrogel supercapacitors are prepared based on the hydrogels, showing high specific capacitance (212.5 mF cm^−2^), excellent energy density (18.89 µWh cm^−2^), and large deformability. Moreover, flexible self‐powered luminescent integrated systems are constructed based on the supercapacitors, which can spontaneously shine anytime and anywhere without extra power. This work provides new insights and feasible avenues for developing high‐performance stretchable electrode materials and energy storage devices for wearable electronics.

## Graphical abstract

This work pioneeringly proposes a distinct confinement self‐assembly and multiple crosslinking strategy to develop a new type of organic–inorganic hybrid conductive hydrogels. The hybrid conductive hydrogels simultaneously exhibit high conductivity, excellent stretchability, and superior electrochemical performance. Stretchable elastic all‐hydrogel supercapacitors and flexible self‐powered luminescent integrated systems have been constructed based on the high‐performance hybrid conductive hydrogels.

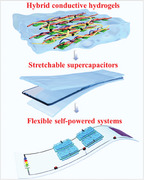

## 1 Introduction

Wearable electronics, as an emerging cutting‐edge technology, have broad application prospects in artificial intelligence, human‐computer interactions, health monitoring systems, etc.^[^ ^1^, ^2^, ^3^ ^]^ Generally, wearable electronic equipments need to operate in highly deformed states to adapt to limb movements, such as muscle stretching and contraction. In this case, they should maintain normal electrical behavior under large deformation and have recently generated great research interest. To satisfy the need of wearable electronic equipments, as their core energy supply element, energy storage devices require both high specific capacity and arbitrary deformability.^[^ ^4^ ^]^ In other words, the ideal energy storage devices that can match well with wearable electronics are supposed to keep high specific capacity not only in bending state but also in twisted, and even in stretched state.^[^ ^5^, ^6^, ^7^, ^8^ ^]^ As a key component of energy storage devices, the electrode has a great influence on device performances. To prepare high‐capacity and largely deformable energy storage devices, the key is the design and exploitation of ideal electrode materials that simultaneously possess high electrical conductivity, superior electrochemical activity, and excellent mechanical stretchability.

Conductive polymer hydrogels, especially poly(3,4‐ethylenedioxythiophene)/poly(styrene‐sulfonate) (PEDOT:PSS) hydrogels,^[^ ^9^, ^10^, ^11^ ^]^ have not only the conductivity and pseudocapacitance of conductive polymers but also the adjustable mechanical flexibility of hydrogels, making them highly competitive electrode materials for deformable and high‐capacity energy storage devices, standing out from various electrode materials. So far, PEDOT:PSS hydrogels are mainly prepared by two methods. The first method is the pure PEDOT:PSS hydrogel prepared by additive‐induced cross‐linking or strong acid‐treated gelation.^[^ ^12^, ^13^, ^14^, ^15^ ^]^ Pure PEDOT:PSS hydrogels usually exhibit high electrical conductivity but limited mechanical stretchability (<60%) due to lack of soft insulating polymer network for stress transfer and energy dissipation. The second approach to prepare PEDOT:PSS hydrogel usually combines the conductive PEDOT:PSS component with a soft insulating polymer network.^[^ ^16^, ^17^, ^18^ ^]^ The introduction of soft insulating networks for stress dissipation and stretchability improvement inevitably deteriorates the connectivity of rigid conductive phase. Thus, the resulting hydrogels exhibit high stretchability but low electrical conductivity (0.1–23 S m^−1^) and electrochemical activity. In brief, design and construction of ideal microstructures in conductive polymer hydrogels that can effectively dissipate stress without obvious influence on the continuity of conductive domains is difficult so far. Therefore, it remains extremely challenging to synergistically regulate the electrical, electrochemical, and mechanical properties of conductive polymer hydrogels by the aforementioned methods, resulting in an extreme scarcity of electrode materials with excellent comprehensive performance, which limits further development of large‐deformable and high‐capacity energy storage devices and wearable electronics.

Considering MXene's high electrical conductivity, high electrochemical activity and abundant surface functional groups for bonding with polymers, it shows great potential in improving the performance of conductive polymer hydrogels. Herein, a distinct confinement self‐assembly and multiple crosslinking strategy has been proposed to develop a new type of organic–inorganic hybrid conductive hydrogels composed of poly(3,4‐ethylenedioxythiophene):poly(styrene sulfonate) (PEDOT:PSS), carboxyl titanium carbide (C‐MXene) and polyvinyl alcohol cross‐linked by glutaraldehyde (GA‐PVA), called after PEDOT:PSS/C‐MXene/GA‐PVA. The confinement self‐assembly and multiple crosslinking effect cause the hydrogels to form a biphase interpenetrating cross‐linked network. The interpenetrating networks can reconfigure along stretching direction for improving mechanical properties without obvious disruption of the connectivity of the entangled conductive phase. Thus, the organic–inorganic hybrid conductive hydrogels exhibit outstanding electrical conductivity (≈2000 S m^−1^), large mechanical stretchability (≈200%), excellent resilience, and high electrochemical activity all at once. A class of stretchable elastic all‐hydrogel supercapacitors (SCs) have been prepared by using the hybrid conductive hydrogels as electrodes, showing high specific capacitance (212.5 mF cm^−2^), superb energy storage capacity (energy density of 18.89 µWh cm^−2^, power density of 400.02 µW cm^−2^), and large deformability (including bendability, twistability and stretchability). Furthermore, flexible self‐powered luminescent integrated systems have been constructed based on the resulting SCs, which can spontaneously shine anytime and anywhere without the need for external power supply. This work provides a feasible technical solution for developing high‐performance flexible/stretchable electronic materials, devices, and integrated systems, laying a foundation for the rapid development of flexible wearable electronics.

## 2 Results and Discussion

### 2.1 Design and Fabrication of Organic–Inorganic Hybrid Conductive Hydrogels

**Figure 1:**
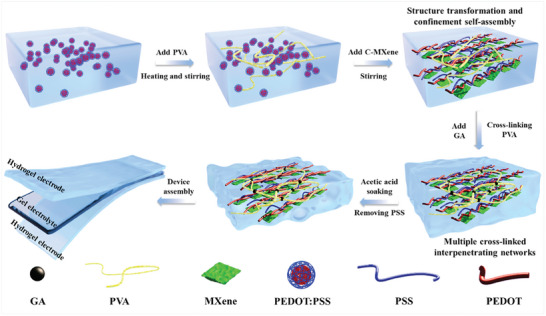
Schematic diagram of the preparation procedures of organic–inorganic hybrid conductive hydrogels and all‐hydrogel supercapacitors.

**Figure** **1** illustrates the preparation process of the organic–inorganic hybrid conductive hydrogels and the all‐hydrogel supercapacitors. In brief, PVA was added into PEDOT:PSS solution followed by heating and stirring to dissolve PVA and achieve PEDOT:PSS/PVA solution. Then, C‐MXene solution was mixed with the aforementioned PEDOT:PSS/PVA solution to induce configuration transformation of PEDOT by interaction and electronic structure change. Subsequently, a small amount of GA was further introduced to cross‐link PVA and contribute to the gelation of the solution. Finally, acetic acid soaking was carried out to eliminate PSS and high‐performance PEDOT:PSS/C‐MXene/GA‐PVA hybrid conductive hydrogel was achieved. All‐hydrogel supercapacitors were assembled by sandwiching a gel electrolyte between the two hybrid conductive hydrogel electrodes. Specific experimental procedures were provided in Supporting Information.

**Figure 2:**
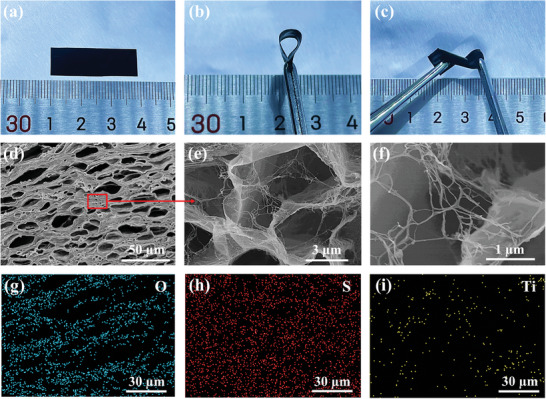
Morphology and structure of the hybrid conductive hydrogels. a–c) Photos of the initial, bended, and twisted hybrid conductive hydrogels. d–f) Cross‐sectional SEM images of hybrid conductive hydrogels. g–i) EDS mapping of hybrid conductive hydrogels.

**Figure** **2**a–c are the photos of the resulting PEDOT:PSS/C‐MXene/GA‐PVA hybrid conductive hydrogels before and after deformations. Scanning electron microscopy (SEM) were used to characterize the microstructure and morphology of original PEDOT:PSS, C‐MXene, and the resulting PEDOT:PSS/C‐MXene/GA‐PVA hybrid conductive hydrogel, as shown in Figure S1a,b (Supporting Information) and Figure 2d–f. Original PEDOT:PSS usually possessed microgel particle structure,^[^ ^12^ ^]^ which could be proved by the magnified SEM image (Figure S1a, Supporting Information). Whereas, C‐MXene exhibited typical 2D nanosheet structure with size of several micrometers (Figure S1b, Supporting Information). By contrast, the hybrid conductive hydrogels possessed a unique layered porous microstructure (Figure 2d–f). As shown in the enlarged images, the structure of PEDOT:PSS transformed from particles to chains and were confined to grow between laminated C‐MXene nanosheets after hybridization with C‐MXenes (Figure 2e,f). The energy dispersive X‐ray spectroscopy (EDS) mapping tests indicated homogeneous distribution of PEDOT:PSS and C‐MXene nanosheets in the hybrid conductive hydrogels (Figure 2f–i).

### 2.2 Mechanical and Electrical Properties of Organic–Inorganic Hybrid Conductive Hydrogels

A compromise between mechanical and electrical properties is often observed in existing PEDOT:PSS hydrogels. As mentioned above, pure PEDOT:PSS hydrogels exhibit low mechanical stretchability without soft insulating polymer networks. However, the introduction of soft insulating network inevitably decreases the content and deteriorates the continuity of conductive component, leading to limited electrical conductivity. A method has been reported to restrain this compromise by enhancing the relative mass ratio of PEDOT:PSS to PVA (≈1:1) following by acid treatment.^[^ ^18^ ^]^ In general, conventional PVA usually exhibits poor structural integrity and low mechanical strength. By contrast, PVA chemically cross‐linked by GA shows excellent mechanical strength, which contributes to improving the mechanical stretchability and elasticity of hydrogels. Thus, GA‐PVA was chosen as the soft insulating polymer network of conductive hydrogels. Using a similar method, we first prepared PEDOT:PSS/GA‐PVA and C‐MXene/GA‐PVA hydrogel based on single conductive materials. Their mechanical stretchability and electrical conductivity were measured. The PEDOT:PSS/GA‐PVA hydrogel showed a stretchability of ≈102% (Figure S2a, Supporting Information) and a conductivity of ≈945 S m^−1^. The C‐MXene/GA‐PVA hydrogel exhibited a stretchability of ≈70% (Figure S2b, Supporting Information) and a conductivity of ≈17 S m^−1^.

**Figure 3:**
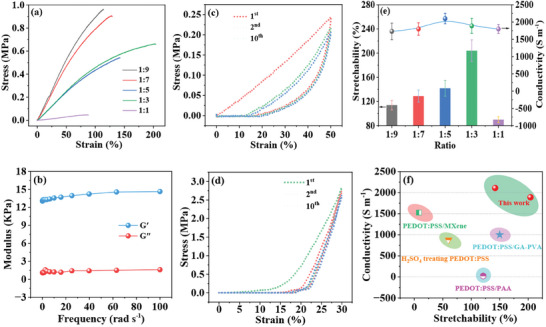
Mechanical and electrical properties of the hybrid conductive hydrogels. a) Stress–strain curves of the hybrid conductive hydrogels with different volume ratios. b) Rheological performance test of the hybrid conductive hydrogel with volume ratio of 1:3. c) Loading–unloading curves of the hybrid conductive hydrogels with volume ratio of 1:3 at 50% tensile strain for ten cycles. d) Loading‐unloading curves of the hybrid conductive hydrogels with volume ratio of 1:3 at 30% compression strain for ten cycles. e) Mechanical stretchability and electrical conductivity of the hybrid conductive hydrogels with different volume ratios. f) Performances comparison of the current work with previous works.

To further improve the comprehensive performances of PEDOT:PSS/GA‐PVA and C‐MXene/GA‐PVA hydrogel, a confinement self‐assembly and multiple crosslinking strategy was created by hybridization of PEDOT:PSS with C‐MXene while keeping the mass ratio of PEDOT:PSS to PVA at 1:1 to prepare organic–inorganic hybrid conductive hydrogels, i.e., PEDOT:PSS/C‐MXene/GA‐PVA. The effect of the amount of C‐MXene on the mechanical and electrical properties of the hybrid conductive hydrogels was systematically investigated. Stretchability and modulus are commonly used to characterize the mechanical properties of hydrogels.^[^ ^19^, ^20^, ^21^, ^22^ ^]^ **Figure** **3**a shows the stress‐strain curves of the as‐prepared hybrid conductive hydrogels by regulating the volume ratio of C‐MXene solution to PEDOT:PSS/GA‐PVA solution. C‐MXene could significantly improve the mechanical performances of the hydrogels. Their stretchability increased gradually with the increment of C‐MXene. When the ratio increased from 1:9 to 1:3, the stretchability could be increased from ≈114% to ≈204%, which was much larger than that of PEDOT:PSS/GA‐PVA and C‐MXene hydrogel. Meanwhile, the elastic modulus was decreased from 1.07 to 0.52 MPa, as illustrated in Figure S3 (Supporting Information). Nevertheless, when the ratio surpassed 1:3, the stretchability of the hybrid conductive hydrogels decreased while the elastic modulus was further decreased to 0.07 MPa. Thus, to achieve optimal mechanical stretchability, the volume ratio was chosen to be 1:3.

The hybrid conductive hydrogels exhibited not only excellent stretchability but also superior elasticity and resilience. To prove their elasticity, rheological property of the hybrid conductive hydrogels with ratio of 1:3 was tested (Figure 3b). The loss modulus (G″) was lower than storage modulus (G′), verifying that the mechanical property of the hybrid hydrogel was dominated by elasticity. Moreover, tensile and compression cycles of the hybrid conductive hydrogels with ratio of 1:3 were recorded to investigate their fatigue resistance. Figure 3c,d illustrate the loading–unloading curves at 50% tensile strain and 30% compression strain for ten cycles, respectively. Although large hysteresis loop was observed in the first cycle, it significantly reduced from the second cycle for both stretch and compression. Notably, the loading‐unloading curves of the subsequent cycles overlapped quite well without obvious residual strain or large hysteresis loop, indicating their excellent fatigue resistance and resilience. Most previously reported elastomers ^[^ ^23^ ^]^ and hydrogels ^[^ ^24^ ^]^ merely showed brilliant stretchability without superior resilience and could not return to the initial shape after stretch. By contrast, our organic–inorganic hybrid conductive hydrogels presented not only high mechanical stretchability but also outstanding elasticity and resilience, resulting in high deformability, durability and reliability.

Besides mechanical properties, the electrical conductivity of the hybrid conductive hydrogels could also be ameliorated by C‐MXene. As shown in Figure 3e, the electrical conductivity of the PEDOT:PSS/C‐MXene/GA‐PVA hydrogel was ≈1700 S m^−1^ when the volume ratio of C‐MXene to PEDOT:PSS/GA‐PVA was 1:9. The electrical conductivity could be enhanced to ≈2100 S m^−1^ when the ratio increased to 1:5. However, it then decreased to ≈1900 and 1800 S m^−1^ when the ratio was further increased to 1:3 and 1:1, respectively. Despite the electrical conductivity increased at first and then decreased with the increase of C‐MXene, it was around 2000 S m^−1^ and was much larger than that of PEDOT:PSS/GA‐PVA and C‐MXene/GA‐PVA hydrogel.

Both the mechanical stretchabiliy and electrical conductivity increased at first and then decreased with the increase of C‐MXene. This could be attributed to the following reasons. The C‐MXene could induce PEDOT to form entangled long chains and physically cross‐linked networks, which not only allowed electron transport but also dissipated stress during the stretching process. Thus, the electrical conductivity and mechanical stretchability improved with the increase of C‐MXene. However, when the amount C‐MXene was too much, aggregation and inhomogeneous distribution occurred, which was adverse to the connection of conductive paths and the dissipation of stress. Thus, conductivity and stretchability then decreased. The mechanism for performance improvement will be discussed in detail in Section 2.3.

On the whole, when the ratio is 1:3, the hybrid conductive hydrogel simultaneously achieves excellent stretchability (≈204%) and high conductivity (≈1900 S m^−1^) (Figure 3e). Currently, pure PEDOT:PSS hydrogels show high conductivity on the order of 1000 S m^−1^ but can only endure small strains (<60%).^[^ ^12^, ^14^ ^]^ Those combined with insulating polymer networks are stretchable (>100%), their conductivity, however, are much lower (0.1–23 S m^−1^).^[^ ^16^, ^17^ ^]^ In comparison, both the excellent electrical and mechanical performances of our hybrid conductive hydrogel are superior to the existing best‐performing conductive hydrogels,^[^ ^14^, ^16^, ^18^, ^25^ ^]^ as illustrated in Figure 3f. Thus, the hybrid conductive hydrogel is promising as electrode for applications in high‐performance deformable energy storage devices and wearable electronics.

### 2.3 Mechanisms for High Mechanical and Electrical Properties

**Figure 4:**
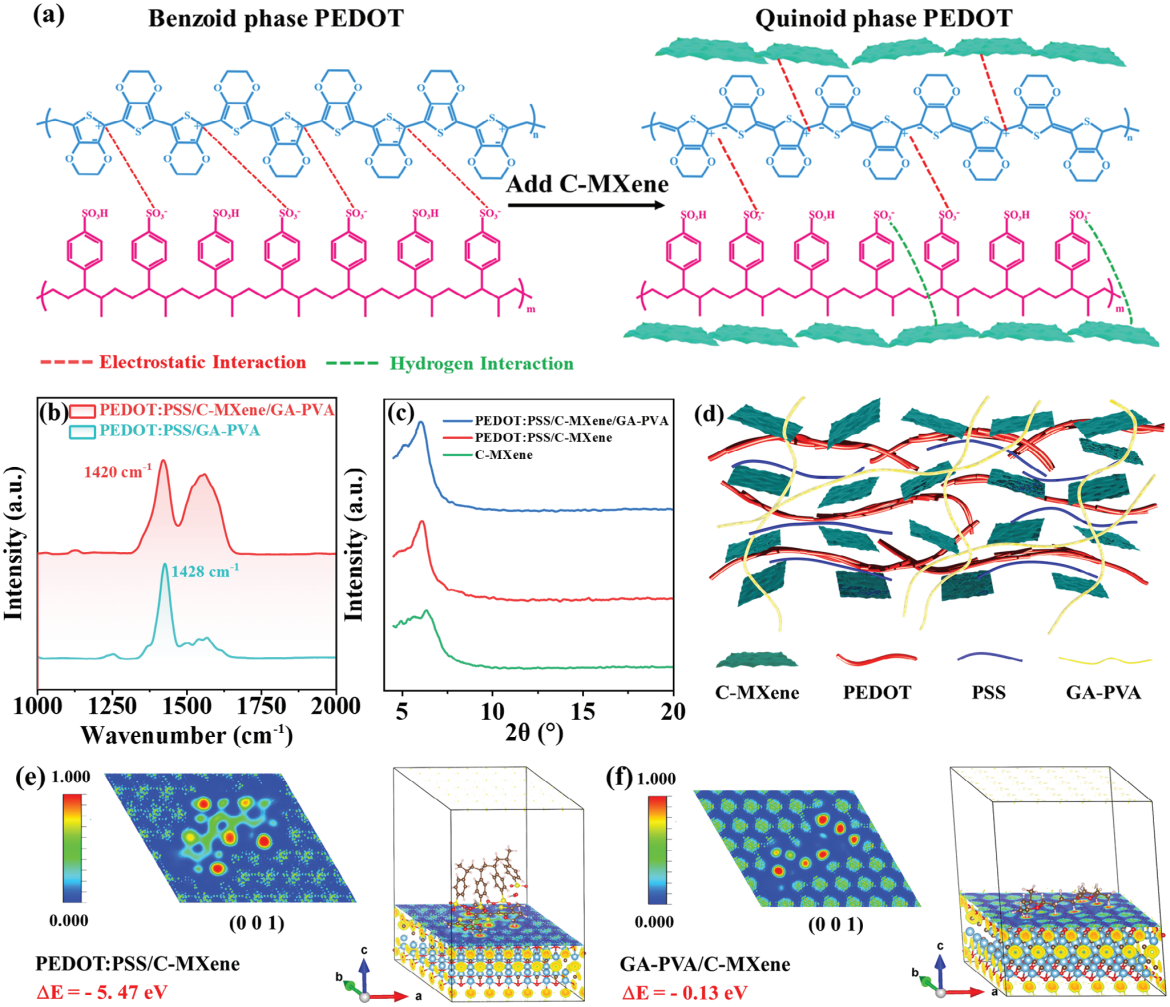
Mechanisms for high mechanical and electrical properties of the hybrid conductive hydrogels. a) Configuration of PEDOT:PSS before and after introducing C‐MXene. b) Raman spectra of PEDOT:PSS/GA‐PVA and PEDOT:PSS/C‐MXene/GA‐PVA. c) XRD spectra of C‐MXene, PEDOT:PSS/C‐MXene and PEDOT:PSS/C‐MXene/GA‐PVA. d) Schematic illustration of the inner structure of the hybrid conductive hydrogels. e,f) First‐principles calculations for the interactions between PEDOT:PSS and C‐MXene as well as GA‐PVA and C‐MXene.

The hybridization with C‐MXene induced the configuration transformation of PEDOT from benzoid structures to quinoid structures, as illustrated in **Figure** **4**a. This could be confirmed by Raman spectra (Figure 4b). The PEDOT:PSS/GA‐PVA spectrum showed a feature peak at 1428 cm^−1^ which corresponded to C_α_ = C_β_ symmetric stretching vibration of the thiophene rings on PEDOT. This feature peak redshifted to 1420 cm^−1^ after the introduction of C‐MXene, indicating structure transformation.^[^ ^25^, ^26^ ^]^ The structure transformation of PEDOT could also be proved by X‐ray photoelectron spectra (XPS), as shown in Figure S4a—c (Supporting Information). All the elements of C‐MXene and PEDOT:PSS could be detected all at once within the PEDOT:PSS/C‐MXene/GA‐PVA hybrid conductive hydrogel (Figure S4a, Supporting Information). For the high‐resolution Ti 2p spectra of the hybrid conductive hydrogel, the characteristic peaks at 455.20 eV and 456.65 eV could be attributed to Ti‐C bond and Ti‐O bond for Ti 2p_3/2_ states, which shifted to higher binding energy by 0.32 eV compared to those of C‐MXene (Figure S4b, Supporting Information). Meanwhile, the characteristic peaks at 163.87 eV (S 2p_3/2_ state) and 164.97 eV (S 2p_1/2_ state) for high‐resolution S 2p spectra of the hybrid conductive hydrogel shifted to lower binding energy by 0.21 eV compared to those of PEDOT:PSS (Figure S4c, Supporting Information). The alteration of binding energy peaks was mainly attributed to the electrostatic interactions between PEDOT and C‐MXene, which could change the electronic structure of PEDOT and cause its structure transformation from benzoid to quinoid structure. Generally, PEDOT with benzoid structure possessed a coiled morphology, while the quinoid structure showed linear morphology. On the one hand, the *π–π* interactions between the linear quinoid PEDOT chains were usually stronger than that between the coiled benzoid PEDOT. On the other hand, the electrostatic interactions between the positively charged PEDOT and the negatively charged C‐MXene as well as the hydrogen bonding between the sulfonate of PSS and the carboxyl of C‐MXene bound PEDOT:PSS between C‐MXene layers, as intuitively shown in Figures 2d–f and 4a,d.

To fully prove that PEDOT:PSS were confined between C‐MXene layers, X‐ray diffraction (XRD) patterns of C‐MXene, PEDOT:PSS/C‐MXene and PEDOT:PSS/C‐MXene/GA‐PVA were systematically characterized (Figure 4c). The main diffraction peak of C‐MXene nanosheets was at 2*θ*  = 6.4°, which corresponded to an interlayer spacing of C‐MXene nanosheets. This diffraction peak shifted downward to 6.1° in PEDOT:PSS/C‐MXene, indicating that the interlayer spacing of C‐MXene nanosheets became larger and the PEDOT:PSS chains self‐intercalated into C‐MXenes due to the hydrogen bonding and the electrostatic interactions between them. However, this diffraction peak of PEDOT:PSS/C‐MXene/GA‐PVA was at 6.04° similar to that of PEDOT:PSS/C‐MXene, implying introducing GA‐PVA had no obvious influence on the interlayer spacing. The GA‐PVA chains were likely to interpenetrate with conductive phase in the hydrogel system (Figure 4d). This was probably because the interactions between C‐MXene and GA‐PVA were weaker than that between C‐MXene and PEDOT:PSS. Fourier transform infrared (FTIR) spectra of GA‐PVA, PEDOT:PSS/GA‐PVA, C‐MXene/GA‐PVA, and PEDOT:PSS/C‐MXene/GA‐PVA hydrogel were characterized, as shown in Figure S4d (Supporting Information). The absorption peak at 3300 cm^−1^ corresponded to the stretching vibration of the incomplete cross‐linked hydroxyl groups in the GA‐PVA hydrogel, which redshifted to 3284 and 3285 cm^−1^ for PEDOT:PSS/GA‐PVA and C‐MXene/GA‐PVA hydrogel, respectively. This was likely because the sulfonate of PSS and carboxyl groups of C‐MXene formed stronger intermolecular hydrogen bonds with the hydroxyl groups of GA‐PVA. However, this absorption peak blue shifted to 3373 cm^−1^ for PEDOT:PSS/C‐MXene/GA‐PVA. This also indicated that the interaction between PEDOT:PSS and C‐MXene, including the electrostatic interactions and the hydrogen bonding were stronger, which restricted their bonding with GA‐PVA. Moreover, the introduction of PEDOT:PSS and C‐MXene suppressed the aggregation of GA‐PVA and weakened the hydroxy‐association strength. Hence, blue shift occurred. First‐principles calculations were further carried out to reveal the interactions between PEDOT:PSS and C‐MXene as well as that between GA‐PVA and C‐MXene (Figure 4e,f; Figure S5, Supporting Information). It was observed that PEDOT:PSS and C‐MXene exhibited more negative interaction energy (−5.47 eV) than that of GA‐PVA and C‐MXene, verifying stronger interactions between PEDOT: PSS and C‐MXene. This contributed to the insertion of PEDOT:PSS chains in between C‐MXene layers.

Both of the stronger π‐π interactions of quinoid PEDOT:PSS and their confinement by C‐MXene nanosheets together caused the aggregation and self‐assembly of the quinoid PEDOT into longer chains, as schematically illustrated in Figure 4d. The longer conductive PEDOT chains were more easily overlapped with each other and could bridge the neighboring C‐MXene nanosheets. Likewise, the C‐MXene nanosheets could also connect the adjacent PEDOT long chains. These jointly contributed to forming interconnected entangled conductive phase and significantly improving the electrical conductivity and electrochemical activity. Additionally, acetic acid immersion could remove insulating PSS and expose more conductive PEDOT, which was also helpful for improving electrical conductivity and electrochemical activity.^[^ ^18^ ^]^

The physical cross‐linking, including entanglement among conductive quinoid PEDOT long chains, electrostatic interactions, and hydrogen bonding between PEDOT:PSS and C‐MXene, combined with the chemical cross‐linking between insulating PVA and GA forms biphase interpenetrating 3D cross‐linked networks (Figure 4d). In biphase interpenetrating 3D cross‐linked networks, the chemically cross‐linked soft insulating phase worked together with the physically cross‐linked conductive phase to transfer and dissipate stress during stretching to enhance mechanical stretchability without obvious destruction of the interconnection of the conductive phase. Therefore, the hybrid conductive hydrogels could achieve both high electrical and mechanical performances.

### 2.4 Preparation and Performances Characterization of All‐Hydrogel SCs

**Figure 5:**
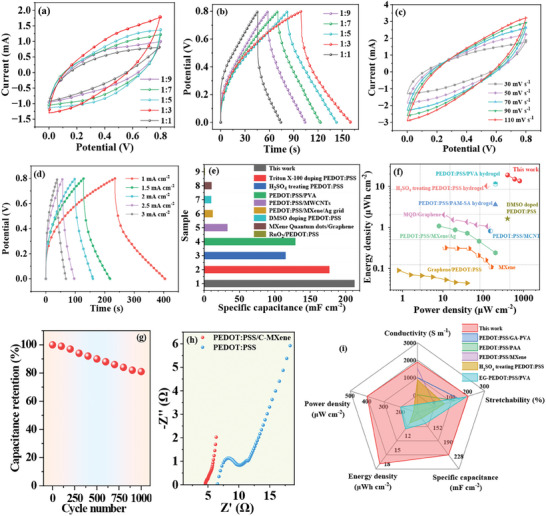
Electrochemical performances and energy storage capacity of the all‐hydrogel SCs. a,b) CV curves (30 mV s^−1^) and GCD curves (2 mA cm^−2^) of the SCs based on the hybrid conductive hydrogels with different volume ratio. c) CV curves at different scan rates of the SCs based on the hybrid conductive hydrogels with a volume ratio of 1:3. d) GCD curves at various current densities of the SCs based on the hybrid conductive hydrogels with a volume ratio of 1:3. e) Specific capacitance comparison of our SCs with others. f) Ragone plots. g) The cycle stability test of the SCs. h) Nyquist plots of the SCs based on PEDOT:PSS/GA‐PVA and PEDOT:PSS/C‐MXene/GA‐PVA. i) Comprehensive performance comparison, including stretchability, conductivity, and electrochemical properties of the hybrid conductive hydrogels in this work with other best‐performing conductive hydrogels.

To investigate the electrochemical performances and demonstrate the feasibility of the resulting hybrid conductive hydrogels for high‐performance energy storage devices, a PVA‐H_3_PO_4_ gel electrolyte was sandwiched between two pieces of hydrogel electrodes to assemble all‐hydrogel SCs. Notably, C‐MXene could also influence the electrochemical performances besides mechanical stretchability and electrical conductivity. The cyclic voltammetry (CV) curves at the scan rate of 30 mV s^−1^ and galvanostatic charge/discharge (GCD) curves at the current density of 2 mA cm^−2^ of the SCs based on the hybrid conductive hydrogels with various volume ratio were systematically recorded (**Figure** **5**a,b). It was discovered that the electrochemical performances improved at first when the volume ratio increased from 1:9 to 1:3. Nonetheless, when the ratio surpassed 1:3 the electrochemical properties of the SCs then declined. The specific capacitance of the SCs based on the hybrid conductive hydrogels with various ratio calculated from the GCD curves (2 mA cm^−2^) was 114.3, 131.3, 151.2, 152.5, and 71.5 mF cm^−2^, respectively. The SC with volume ratio of 1:3 achieved the highest specific capacitance. To prove the outstanding advantages of the PEDOT:PSS/C‐MXene/GA‐PVA hybrid conductive hydrogel, SCs based on PEDOT:PSS/GA‐PVA hydrogel and C‐MXene/GA‐PVA were also fabricated. Their GCD curves at 2 mA cm^−2^ were measured (Figure S6a,b, Supporting Information), and the corresponding specific capacitance was about 65.0 and 64.8 mF cm^−2^, respectively. The specific capacitances of the SCs based on PEDOT:PSS/C‐MXene/GA‐PVA hybrid conductive hydrogels were significantly higher than those of the SC based on the PEDOT:PSS/GA‐PVA and C‐MXene/GA‐PVA hydrogel (Figure S6c, Supporting Information).

Taking the stretchability, conductivity, and electrochemical performances all into consideration, the volume ratio of 1:3 was optimal for achieving the best comprehensive performances. Thus, 1:3 was chosen below. Three‐electrode tests are commonly used to deeply study the electrochemical property of electrode materials.^[^ ^27^ ^]^ Hence, the CV curves at different scan rates and GCD curves at various current densities of the hybrid conductive hydrogel with ratio of 1:3 were recorded by three‐electrode tests, as shown in Figure S7a,b (Supporting Information). The specific capacitance at the current density of 1 mA cm^−2^ was as high as 303.6 mF cm^−2^, demonstrating excellent energy storage capability. Then, CV curves and GCD curves of an all‐hydrogel device based on the hybrid conductive hydrogels were systematically tested, as shown in Figure 5c,d. The specific capacitance calculated from the GCD curve at the current density of 1 mA cm^−2^ was 212.5 mF cm^−2^, which was much higher than that of the existing best‐performing PEDOT:PSS and MXene based SCs (Figure 5e). It was ≈200 folds that of the supercapacitor based on RuO_2_/PEDOT:PSS hybrid electrode (1.06 mF cm^−2^),^[^ ^28^ ^]^ ≈1.85 folds that of the supercapacitor achieved by using H_2_SO_4_ treated PEDOT:PSS (115 mF cm^−2^),^[^ ^14^ ^]^ ≈21.3 folds that of the supercapacitor prepared by using PEDOT:PSS doped with DMSO (10 mF cm^−2^),^[^ ^29^ ^]^ ≈6.5 folds that of the supercapacitor based on the PEDOT:PSS/multiwalled carbon nanotube (32.9 mF cm^−2^),^[^ ^30^ ^]^ ≈1.2 folds that of the supercapacitor on the basis of PEDOT:PSS doped with Triton X‐100 (177 mF cm^−2^),^[^ ^31^ ^]^ ≈20.4 folds that of the supercapacitor based on MXene quantum dots/graphene (10.42 mF cm^−2^),^[^ ^32^ ^]^ and ≈17.7 folds that of the device based on PEDOT:PSS/MXene/Ag grid (12 mF cm^−2^).^[^ ^33^ ^]^

The SCs based on the hybrid conductive hydrogels also demonstrated excellent energy storage capacity. The Ragone plot is presented in Figure 5f. The largest energy density (*E*) of our SCs was 18.89 µWh cm^−2^ while the power density (*P*) was 400.02 µW cm^−2^. The energy density could also reach 13.56 µWh cm^−2^ when the power density was as high as 800.26 µW cm^−2^. The brilliant energy storage capacity of all‐hydrogel SCs was superior to the previously reported best‐performing PEDOT:PSS, MXene and their hybrids‐based SCs, as shown in Table S1 (Supporting Information). Additionally, the device exhibited good cycle stability. Its capacitance retention was still more than 81% after 1000 charge–discharge cycles at a high current density of 6 mA cm^−2^ (Figure 5g).

The reasons for the large specific capacitance and superior energy storage capacity of the SCs based on the hybrid conductive hydrogels were mainly ascribed to the superb electrical conductivity, high electrochemical activity, and the unique layered porous microstructures of PEDOT:PSS/C‐MXene/GA‐PVA. The high conductivity was beneficial to electron transfer and transmission, while the layered porous microstructures were good for ion diffusion, jointly contributing to the excellent energy storage capability. To further confirm the inherent reasons for the superior capacitive behaviors, electrochemical impedance spectra (EIS) of the SCs based on PEDOT:PSS/C‐MXene/GA‐PVA and PEDOT:PSS/GA‐PVA hydrogel were measured, as shown in Figure 5h. In high‐frequency region, the presence of an impedance arc in the SC based on PEDOT:PSS/GA‐PVA hydrogel indicated the existence of charge transfer resistances. In comparison, the SC based on PEDOT:PSS/C‐MXene/GA‐PVA hybrid conductive hydrogels showed no obvious impedance arc. This demonstrated the device possessed lower charge transfer resistances and PEDOT:PSS/C‐MXene/GA‐PVA exhibited intrinsic higher electrical conductivity as well as superior electrochemical activity. Furthermore, the equivalent series resistance of the SC based on PEDOT:PSS/C‐MXene/GA‐PVA was lower than that of the SC based on PEDOT:PSS/GA‐PVA. Meanwhile, the slope in the low‐frequency region of the SC based on PEDOT:PSS/C‐MXene/GA‐PVA got steeper and closer to vertical compared with that of the SC based on PEDOT:PSS/GA‐PVA hydrogel. This demonstrated that the SC based on PEDOT:PSS/C‐MXene/GA‐PVA hybrid conductive hydrogels possessed better capacitance performance and lower ion diffusion resistance because the layered porous microstructures of the hybrid conductive hydrogels provided more ion transport channels. Taken together, the organic–inorganic hybrid conductive hydrogels exhibited extraordinary comprehensive performances in terms of electrical conductivity, mechanical stretchability, and electrochemical activity, surpassing most previously reported best‐performing conducting hydrogels, ^[^ ^14^, ^16^, ^18^, ^25^, ^34^ ^]^ as shown in Figure 5i and Table S2 (Supporting Information).

Moreover, the resulting all‐hydrogel SCs also possessed outstanding elasticity and arbitrary deformability. They could not only be bent, twisted but also stretched. The CV curves were measured when the all‐hydrogel SCs suffered various large deformation. As shown in Figure S8a,b (Supporting Information), the CV curves showed no obvious changes when the SCs were either bent to 180° or highly twisted. More interestingly, their CV curves still remained nearly constant when they were stretched to 30% and 50% strains (Figure S8c, Supporting Information). The excellent deformability of the all‐hydrogel SCs was primarily attributed to the high stretchability and excellent elasticity of the hybrid conductive hydrogel electrodes as well as the strong interface adhesion between gel electrolytes and hydrogel electrodes. To prove the strong adhesion, load test on a device was carried out, as schematically shown in Figure S9a (Supporting Information). One electrode of an all‐hydrogel SC could easily support a weight of 500 g without delamination between hydrogel electrodes and gel electrolytes (Figure S9b, Supporting Information). The 3D interpenetrating networks of the hybrid conductive hydrogels could reconfigure in the direction of stretching while remaining interconnection without significant influence on the conductivity and electrochemical properties. Meanwhile, strong adhesion between the gel electrolytes and hydrogel electrodes could constrain interlayer slip during stretching to ensure normal diffusion and transmission of the electrolyte ions to electrodes. These factors together contributed to the excellent deformability of the all‐hydrogel SCs.

**Figure 6:**
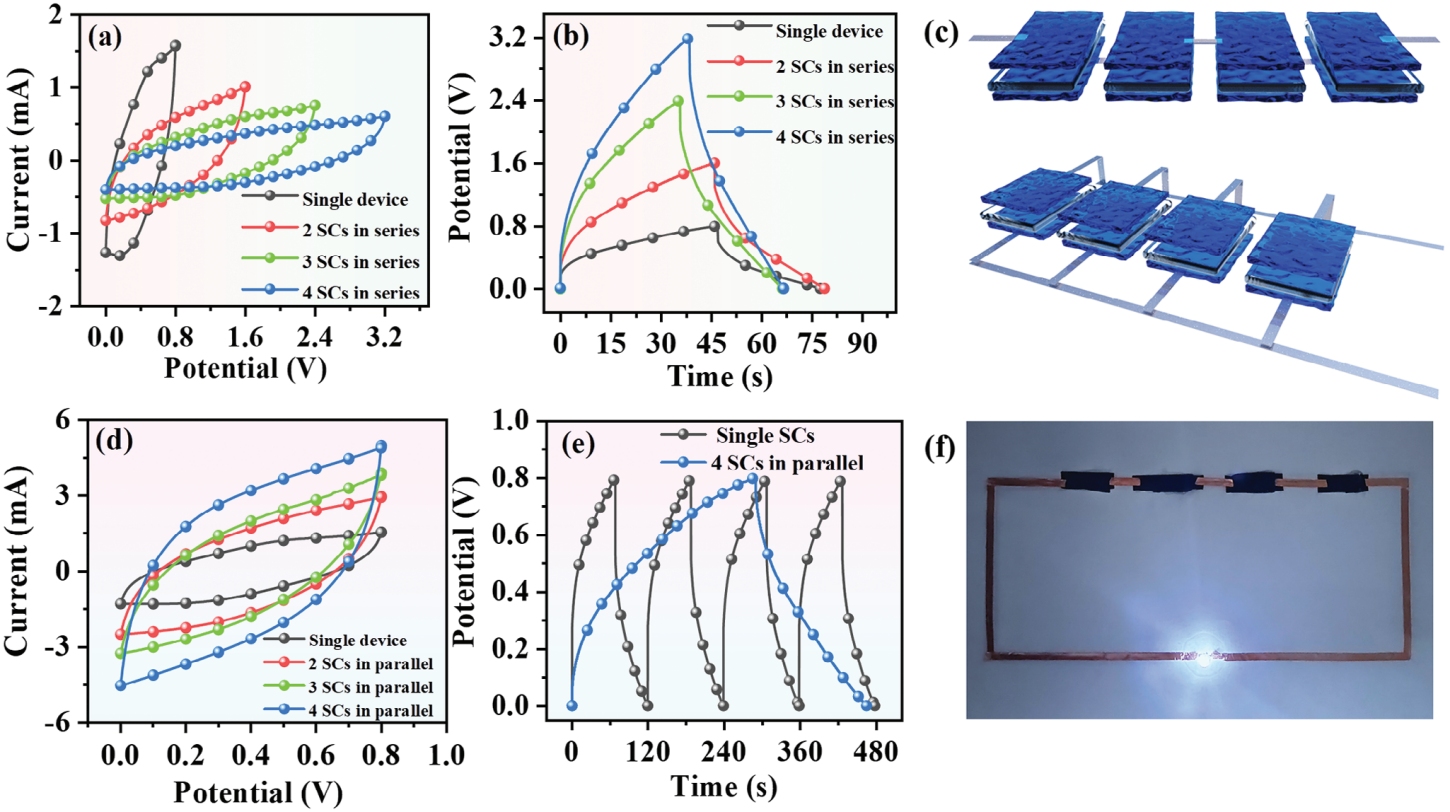
a,b) CV and GCD curves of a single SC and 2–4 SCs connected in series. c) Schematic illustration of four SCs connected in series (top) and in parallel (down). d) CV curves of a single SC and 2–4 SCs connected in parallel. e) GCD curves of a single SC and 4 SCs connected in parallel. f) A LED powered by four SCs connected in series.

Energy storage modules composed of several SCs connected in series or parallel were constructed to tune the capacitance and voltage to enhance the practicability. **Figure** **6**c illustrates four SCs connected in series (top) and parallel (down). Figure 6a,b show the CV and GCD curves of a single SC and 2–4 SCs connected in series. It was observed that the voltage window of the series SCs gradually increased. The voltage of four SCs in series was extended to 3.2 V, which was quadruple that of the single device (0.8 V). In contrast, the current and capacitance of the SCs connected in parallel progressively increased, as proved by the gradually enlarged closed area of the CV curves without changing the voltage window (Figure 6d). The capacitance of the four parallel SCs was approximately fourfolds that of the single one. The amplified capacitance of the parallel SCs could also be validated by discharging longer time as depicted in GCD curves (Figure 6e). The four SCs connected in series could power a blue light‐emitting diode (LED), as shown in Figure 6f, suggesting great potential for practical applications.

### 2.5 Flexible Self‐Powered Luminescent Integrated Systems

The rapid development of human–machine interactions, artificial intelligence, and personal health monitoring system have spurred future electronic devices to be portable, lightweight, wearable, smart, and capable of operation anytime and anywhere without external cumbersome power supplies. Flexible self‐powered integrated system that integrates energy harvesters, energy storage devices, and energy‐consuming devices together on a single flexible substrate is widely considered a viable approach to achieve the aforementioned requirements.^[^ ^35^, ^36^, ^37^ ^]^

**Figure 7:**
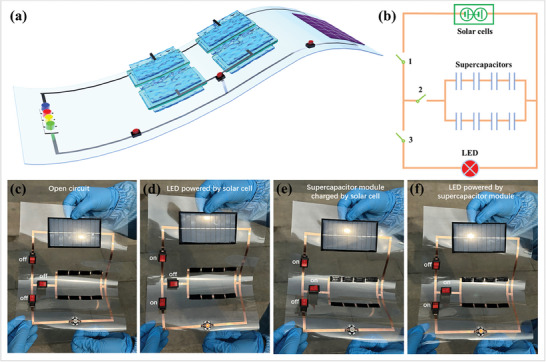
Flexible self‐powered integrated systems. a) Schematic illustration of a flexible self‐powered LED luminescent system. b) The corresponding circuit diagram of the flexible self‐powered LED luminescent system. c) All the switches are off and the system is open circuit. d) LED powered by silicon solar cell. e) Supercapacitor module charged by silicon solar cell. f) LED powered by supercapacitor module.

A flexible self‐powered luminescent integrated system was developed by integrating flexible silicon solar cells, stretchable supercapacitor module and a LED on a soft polyethylene terephthalate (PET) substrate, as schematically illustrated in **Figure** **7**a. The corresponding circuit diagram is shown in Figure 7b. When all switches were off, no power was supplied for the LED, which was not bright (Figure 7c). When switch 1 and 3 were on while switch 2 was off, the silicon solar cells could convert light into electrical energy to power the LED, which could shine (Figure 7d). When switch 1 and 2 were on while switch 3 was off, the silicon solar cells could convert light into electrical energy to charge the supercapacitor module (Figure 7e). Significantly, the charged supercapacitor module was capable of supplying power to the LED to give out light even when switch 1 was off without the assistance of silicon solar cells (Figure 7f). Such a self‐powered integrated system can convert light into electrical energy through solar cells and instantly store the electrical energy in the supercapacitor module to be used at night without the need for extra power, which opens up a new direction in development of future electronics.

## 3 Conclusion

This work ingeniously utilizes a confinement self‐assembly and multiple crosslinking strategy to develop a new class of organic–inorganic hybrid conductive hydrogels. Benefiting from the confinement self‐assembly and multiple crosslinking effect, a 3D interpenetrating conductive network can be formed in the hybrid conductive hydrogels, which is desirable for synergistically regulating the electrical, mechanical, and electrochemical performances. Thus, the hybrid conductive hydrogels simultaneously exhibit superb electrical conductivity (2000 S m^−1^), high mechanical stretchability (200%), and superior electrochemical activity. The unparalleled comprehensive performance of the hybrid conductive hydrogels contributes the subsequent supercapacitors to showing high specific capacitance (212.5 mF cm^−2^), excellent energy density of 18.89 µWh cm^−2^, and large deformability, standing out from the existing various energy storage devices. More importantly, flexible self‐powered luminescent integrated systems have been constructed based on the resulting supercapacitors, which can spontaneously shine at night without the assistance of external power, opening up a new paradigm in electronics. This work not only solves a long‐lasting bottle‐neck issue in developing high‐performance conductive hydrogels with simultaneously ultra‐high electrical conductivity, excellent mechanical stretchability and high electrochemical activity, but also offers a feasible unique path toward next‐generation large‐capacity and largely deformable power sources for extensive applications in multifarious flexible self‐powered integrated systems and intelligent wearable electronics.

## Conflict of Interest

The authors declare no conflict of interest.

## Supporting information

Supporting Information

## Data Availability

The data that support the findings of this study are available in the supplementary material of this article.
