# Supplementary material for: High‐Performance Organic–Inorganic Hybrid Conductive Hydrogels for Stretchable Elastic All‐Hydrogel Supercapacitors and Flexible Self‐Powered Integrated Systems
Source: Adv Sci (Weinh). 2024 Jul 8;11(34):2403358. doi: 10.1002/advs.202403358 (PMC11425858; doi:10.1002/advs.202403358)
Supplement: Supplementary file 1 — Supporting Information [file ADVS-11-2403358-s001.pdf]

ADVANCED SCIENCE
Open Access

Supporting Information

for *Adv. Sci.*, DOI 10.1002/advs.202403358

High-Performance Organic–Inorganic Hybrid Conductive Hydrogels for Stretchable Elastic All-Hydrogel Supercapacitors and Flexible Self-Powered Integrated Systems

Tao Cheng, Zhong-Ting Liu, Jie Qu, Chao-Fu Meng, Ling-Jun He, Lang Li, Xuan-Li Yang, Yu-Jie Cao, Kai Han, Yi-Zhou Zhang and Wen-Yong Lai**

Supporting Information

High-Performance Organic-Inorganic Hybrid Conductive Hydrogels for Stretchable Elastic All-Hydrogel Supercapacitors and Flexible Self-Powered Integrated Systems

Tao Cheng[1,†], Zhong-Ting Liu[1,†], Jie Qu[1], Chao-Fu Meng[1], Ling-Jun He[1,], Lang Li[1], Xuan-Li Yang[1], Yu-Jie Cao[1], Kai Han[1], Yi-Zhou Zhang[2], and Wen-Yong Lai[1,*]*

[1] State Key Laboratory of Organic Electronics and Information Displays (SKLOEID), Institute of Advanced Materials (IAM), School of Chemistry and Life Sciences, Nanjing University of Posts & Telecommunications, 9 Wenyuan Road, Nanjing 210023, China

[2] Institute of Advanced Materials and Flexible Electronics (IAMFE), School of Chemistry and Materials Science, Nanjing University of Information Science & Technology, Nanjing 210044, China

† These authors contributed equally to this work.

* Correspondence author. Email: iamwylai@njupt.edu.cn

Experimental Section

Materials: All reagents are used as received without further purification. C-MXene aqueous dispersion (Ti_3C_2Tx-COOH, 10 mg mL^{-1}) was purchased from Beike Nanomaterial Technology Co., LTD. PEDOT:PSS (Clevios PH1000, 1.3 wt%) was purchased from Haraeus Electronic Materials. Polyvinyl alcohol (M_w 146000-186000) was purchased from Sigma-Aldrich. Glutaraldehyde solution (25%, GA) was purchased from Aladdin. Acetic acid (GR, 99.8%) was purchased from Aladdin and H_3PO_4 was purchased from Shanghai McLean Biochemical Co., LTD.

Preparation of PEDOT:PSS/C-MXene/GA-PVA hydrogel: 0.1 g PVA powder and 7.7 g PEDOT:PSS solution were added to a reagent bottle. The mixture was heated and stirred at 90°C for 6 h until the PVA was dissolved to obtain PEDOT:PSS/PVA solution. After the PEDOT:PSS/PVA solution was cooled to room temperature, a certain volume of C-MXene solution was added to it and stirred for 20 min. Then, a small amount of GA solution was further added and continuously stirred to obtain the precursor solution followed by transferring to a mold and vacuum drying for 6 h. The GA cross-linked hydrogel was then soaked in acetic acid for 6 h and then thoroughly rinsed in water to form PEDOT:PSS/C-MXene/GA-PVA hydrogel.

Assembly of the all-hydrogel supercapacitors: 1.0 g H_3PO_4 and 1.0 g PVA powder were added to 10.0 mL deionized water, which was then heated and stirred at 90°C until turning into gel state to prepare gel electrolyte. The gel electrolyte was then sandwiched between two hybrid hydrogel electrodes to prepare SCs.

Morphology, structure and performance characterization: Scanning electron microscopy was used to characterize the morphology and microstructure of the hydrogels. X-ray diffraction (Bruker D8 Adv) was used to investigate the crystal structure of the samples. FTIR spectra were characterized using a Nicolet iS 50 FTIR spectrometer. Raman spectra were collected using a Laser confocal Raman

spectroscopy (Horba Scientific LabRAM HR Evolution). XPS spectrometer (Kratos Axis Supra) was used to analyze the chemical composition of the samples. A standard four-point probe was used to measure the sheet resistance of the hydrogel electrode. The conductivity was calculated according to the following formula:

$$\delta = \frac{1}{R_s T}$$

In the above formula, δ is the conductivity, R_s is the sheet resistance, and T is the thickness of the hydrogel film. The stress-strain curves, tensile cycle curves and compression cycle curves of the hydrogel were recorded by INSTRON 3343 universal tensile testing machine. The storage modulus (G') and loss modulus (G") were measured by a DHR rheometer. Electrochemical characterization was performed by CHI660 electrochemical workstation. The areal specific capacitance (*C*), power density (*P*) and energy density (*E*) of the SCs were calculated according to the following formulations:

$$C = \frac{I\Delta t}{S\Delta V}$$

$$E = \frac{1}{2} C\Delta V^2$$

$$P = \frac{E}{\Delta t}$$

where I is the discharge current; Δt is the discharge time; S is the effective area and ΔV is the voltage window.

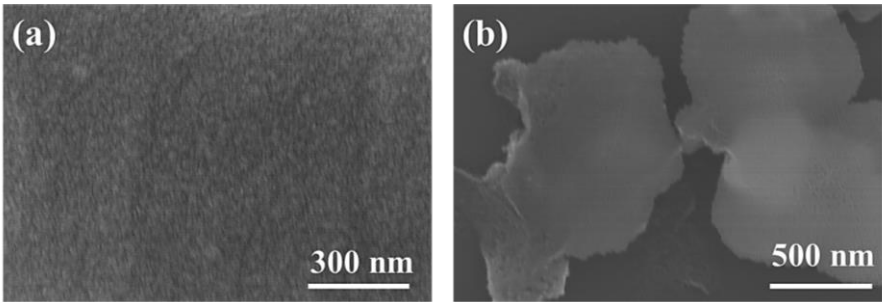

Figure S1. a-b) SEM images of single PEDOT:PSS and C-MXene, respectively.

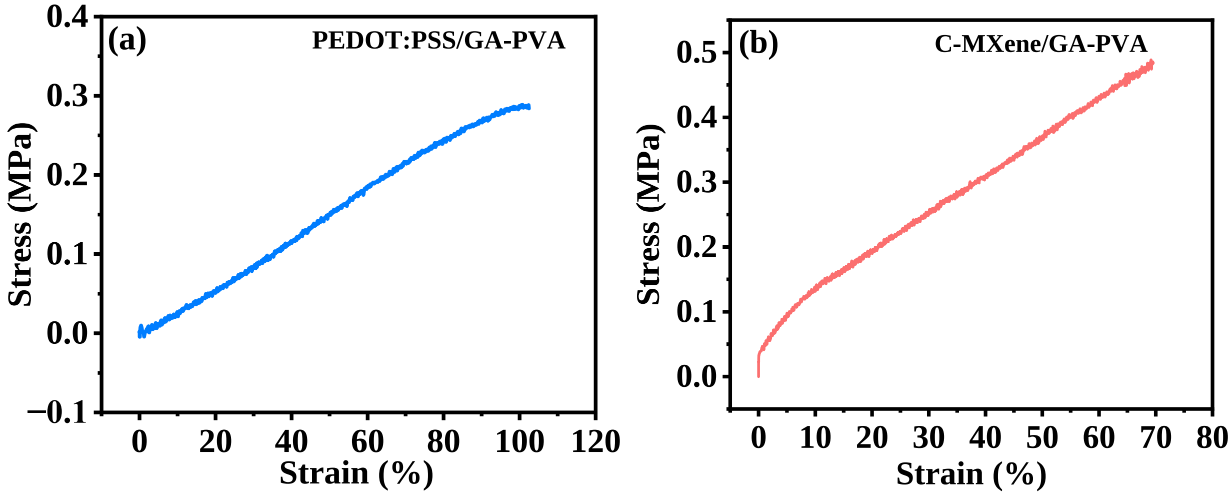

Figure S2. a-b) Stress-strain curves of PEDOT:PSS/GA-PVA and C-MXene/GA-PVA hydrogel.

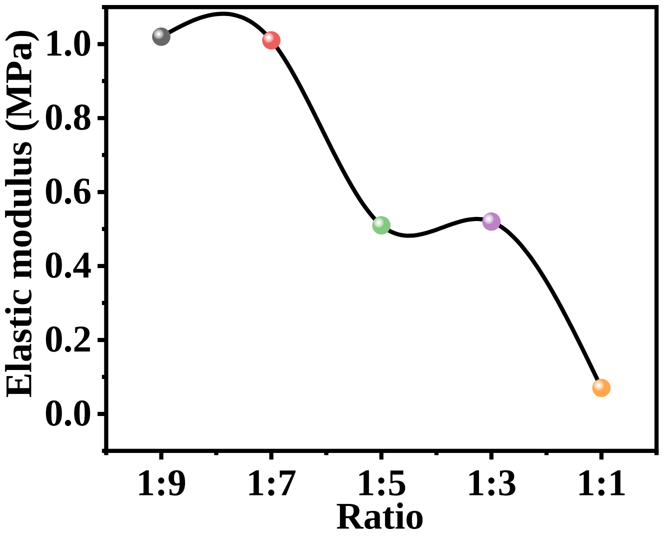

Figure S3. Elastic modulus of the hybrid conductive hydrogels with different volume ratios.

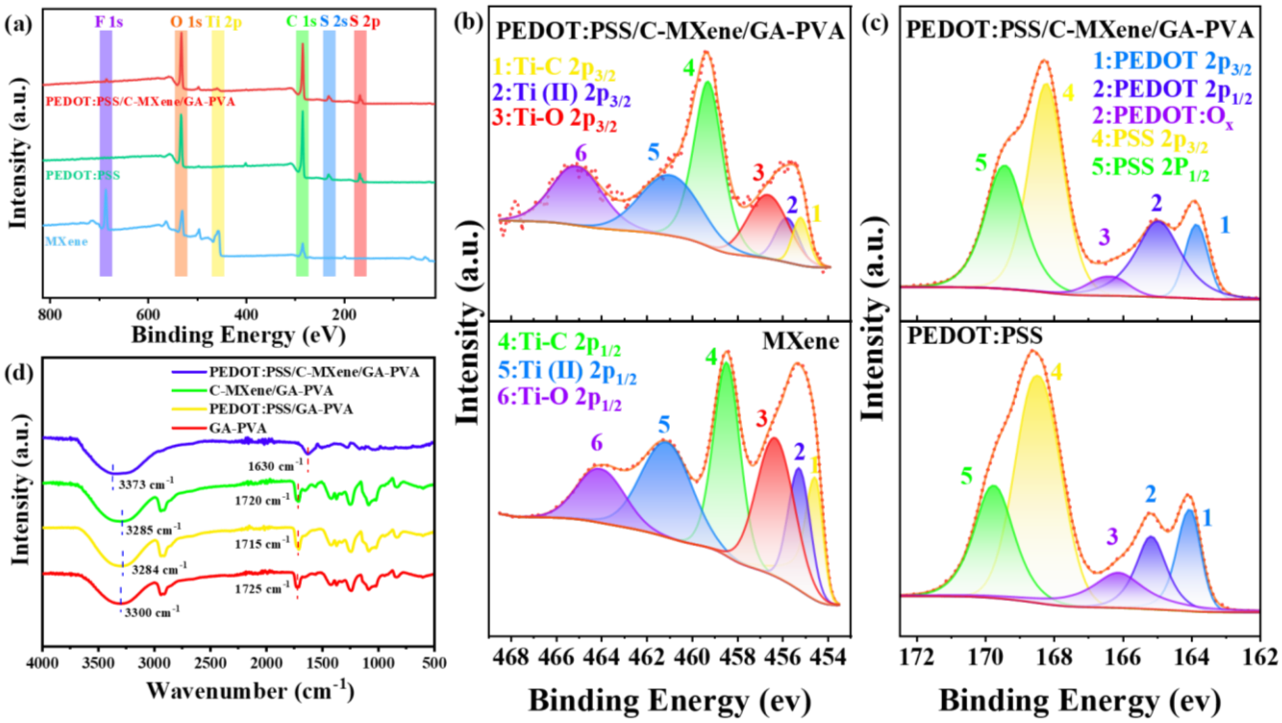

Figure S4. a-c) All elements, Ti 2p, S 2p of PEDOT:PSS, MXene, and PEDOT:PSS/C-MXene/GA-PVA hybrid hydrogel, respectively. d) FTIR spectra of GA-PVA, PEDOT:PSS/GA-PVA, C-MXene/GA-PVA, and PEDOT:PSS/C-MXene/GA-PVA hydrogel, respectively.

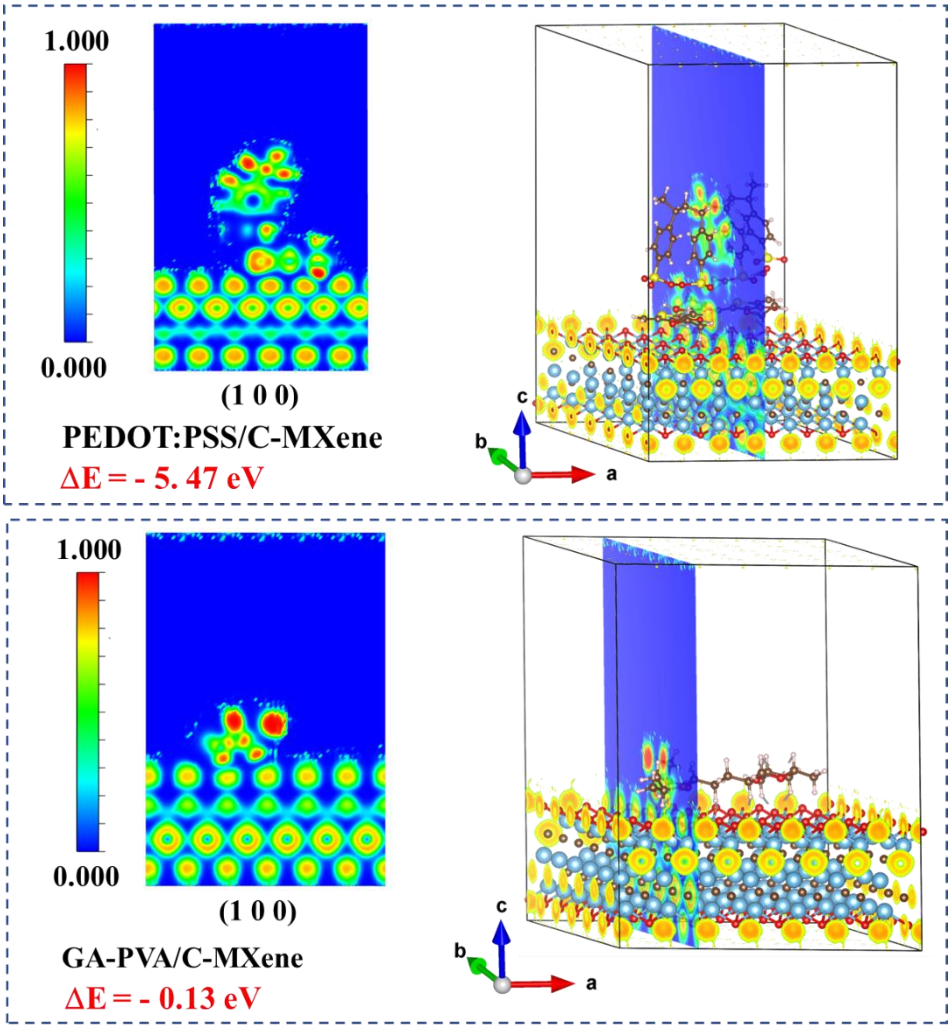

Figure S5. First-principles calculations for interactions between PEDOT:PSS and C-MXene as well as GA-PVA and C-MXene.

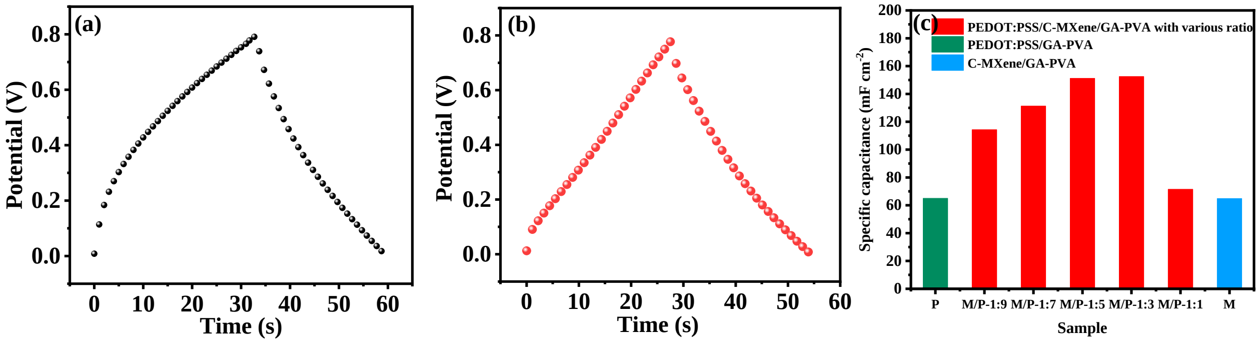

Figure S6. (a) and (b) GCD curves at current density of 2 mA cm^{-2} of SCs based on PEDOT:PSS/GA-PVA and C-MXene/GA-PVA hydrogels, respectively. (c) Specific capacitances of the SCs based on C-MXene/GA-PVA hydrogel, PEDOT:PSS/GA-PVA hydrogel and PEDOT:PSS/C-MXene/GA-PVA hydrogels with different volume ratios.

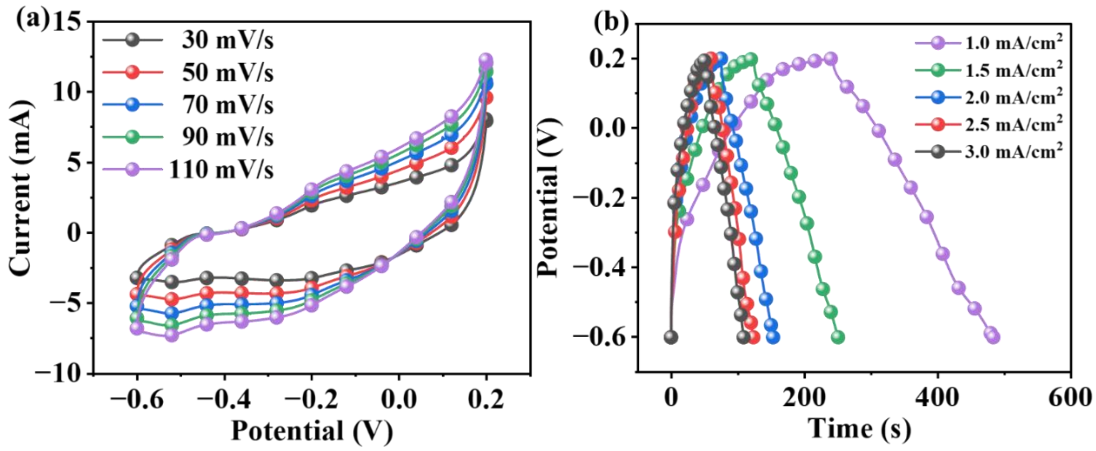

Figure S7. Three electrode test of the hybrid conductive hydrogels with a volume ratio of 1:3. a) CV curves at different scan rates. b) GCD curves at various current densities.

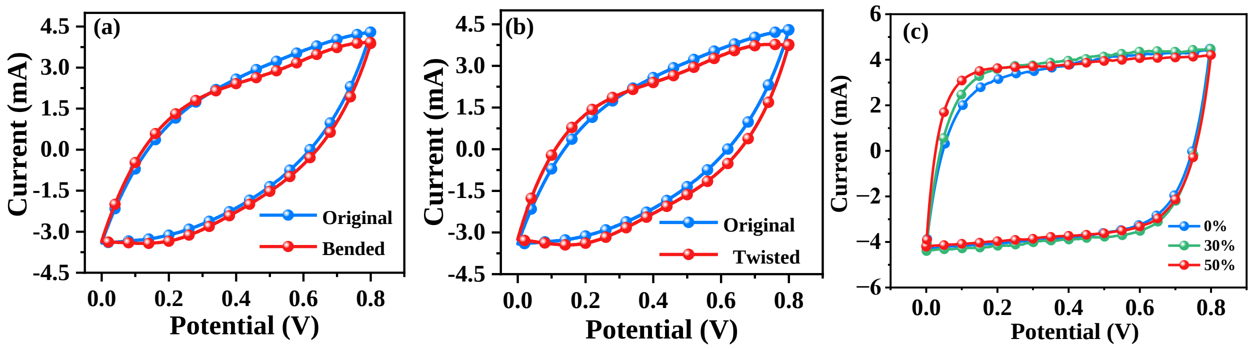

Figure S8. CV curves of all-hydrogel SCs before and after various deformations. a) Original and bended state. b) Original and twisted state. c) Original and stretched to 30% and 50%.

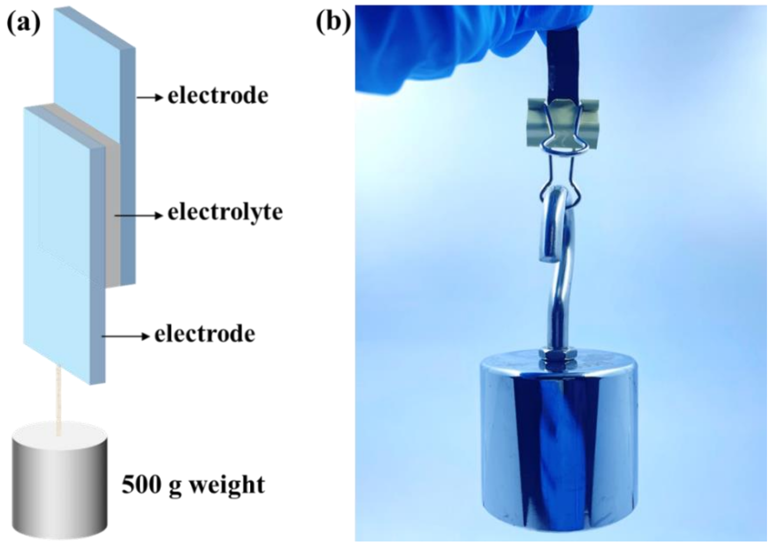

Figure S9. a) Schematic illustration of load test on a device b) Photograph of a SC supporting a weight of 500 g.

Table S1. Energy density & power density of the current work in comparison to others.

Reference	Electrode materials	Power density (μW cm^{-2})	Energy density (μWh cm^{-2})
This work	PEDOT:PSS/C-MXene/GA-PVA hydrogel	400.02<br>600<br>800.26	18.89<br>15<br>13.56
Ref[1]	H_2SO_4 treating PEDOT:PSS hydrogel	122.94	10.22
Ref[2]	PEDOT:PSS/MXene/Ag	7.98<br>20.1<br>40.25<br>78.86<br>200.93	1.08<br>0.87<br>0.72<br>0.46<br>0.24
Ref[3]	MXene Quantum Dots/Graphene	10.92<br>18.4<br>40.8<br>69.6<br>129.4	2.04<br>1.54<br>1.36<br>1.16<br>1.07
Ref[4]	PEDOT:PSS/PAM-SA hydrogel	200	3.6
Ref[5]	DMSO doped PEDOT:PSS	400	1.63
Ref[6]	PEDOT:PSS/ferritin/multiwalled carbon nanotube	150	0.82
Ref[7]	Graphene/PEDOT:PSS	0.8<br>1.6<br>3<br>5.6<br>12<br>20.8<br>40	0.09<br>0.07<br>0.067<br>0.062<br>0.053<br>0.046<br>0.044
Ref[8]	PEDOT:PSS/PVA hydrogel	200.5	11.46
Ref[9]	MXene	11.4<br>22.5<br>44.1<br>75.1<br>115.4<br>157.7	0.32<br>0.31<br>0.307<br>0.209<br>0.16<br>0.109

Table S2. Comprehensive performance of the current work in comparison to others.

**References**	**Electrical conductivity (S m^{-1})**	**Stretchability (%)**	**Specific capacitance (mF cm^{-2})**	**Energy density (µWh cm^{-2})**	**Power density (µW cm^{-2})**
This work	1891	202	212.5	18.89	400.02
PEDOT:PSS/GA-PVA hydrogel	1000	150	/	/	/
PEDOT:PSS/PAA hydrogel	23	121	/	/	/
PEDOT:PSS/MXene hydrogel	1525.8	8	/	/	/
H_2SO_4 treating PEDOT:PSS hydrogel	880	60	115	10.22	122.94
EG-PEDOT:PSS/PVA	/	190.2	128.9	11.46	200.5

References

[1] B. Yao, H. Wang, Q. Zhou, M. Wu, M. Zhang, C. Li, G. Shi, *Adv. Mater.* **2017**, *29*, 1700974.

[2] T. Cheng, X.-L. Yang, S. Yang, L. Li, Z.-T. Liu, J. Qu, C.-F. Meng, X.-C. Li, Y.-Z. Zhang, W.-Y. Lai, *Adv. Funct. Mater.* **2023**, *33*, 2210997.

[3] Y. Yuan, L. Jiang, X. Li, P. Zuo, X. Zhang, Y. Lian, Y. Ma, M. Liang, Y. Zhao, L. Qu, *Adv. Mater.* **2022**, *34*, 2110013.

[4] J. Zeng, L. Dong, W. Sha, L. Wei, X. Guo, *Chem. Eng. J.* **2020**, *383*, 123098.

[5] L. Manjakkal, A. Pullanchiyodan, N. Yogeswaran, E. S. Hosseini, R. Dahiya, *Adv. Mater.* **2020**, *32*, 1907254.

[6] H. J. Sim, C. Choi, D. Y. Lee, H. Kim, J.-H. Yun, J. M. Kim, T. M. Kang, R. Ovalle, R. H. Baughman, C. W. Kee, S. J. Kim, *Nano Energy* **2018**, *47*, 385.

[7] Z. Liu, Z.-S. Wu, S. Yang, R. Dong, X. Feng, K. Muellen, *Adv. Mater.* **2016**, *28*, 2217.

[8] Q. Liu, J. Qiu, C. Yang, L. Zang, G. Zhang, E. Sakai, *Adv. Mater. Technol.* **2021**, *6*, 2000919.

[9] C. Zhang, L. McKeon, M. P. Kremer, S.-H. Park, O. Ronan, A. Seral-Ascaso, S. Barwich, C. O. Coileain, N. McEvoy, H. C. Nerl, B. Anasori, J. N. Coleman, Y. Gogotsi, V. Nicolosi, *Nat. Commun.* **2019**, *10*, 1795.
